# Dyslipidemia Diagnosis and Treatment: Risk Stratification in Children and Adolescents

**DOI:** 10.1155/2022/4782344

**Published:** 2022-02-21

**Authors:** Sara Mosca, Graça Araújo, Vanessa Costa, Joana Correia, Anabela Bandeira, Esmeralda Martins, Helena Mansilha, Mónica Tavares, Margarida P. Coelho

**Affiliations:** ^1^Pediatrics Department, Centro Materno-Infantil do Norte, Centro Hospitalar Universitário do Porto, Porto 4050-651, Portugal; ^2^Pediatrics Department, Hospital Central do Funchal, Funchal 9000-177, Portugal; ^3^Reference Center for Inherited Metabolic Disorders, Centro Materno-Infantil do Norte, Centro Hospitalar Universitário do Porto, Porto 4050-651, Portugal; ^4^Pediatric Nutrition Unit, Pediatrics Department, Centro Materno-Infantil do Norte, Centro Hospitalar Universitário do Porto, Porto 4050-651, Portugal

## Abstract

Dyslipidemias or dyslipoproteinemias are quantitative changes in total cholesterol concentration, respective fractions, or triglycerides in the plasma. Evidence supported that dyslipidemia in childhood is associated with atherosclerosis in adulthood, and early identification and treatment potentially reduce cardiovascular risk in adulthood, which is the principal cause of morbidity and mortality in developed countries. Dyslipidemias can result from primary lipoprotein metabolism changes due to different genetic causes (primary dyslipidemias) or as a consequence of exogenous factors or other pathologies (secondary dyslipidemias). Therefore, the combined dyslipidemias result from the association of important epigenetic and environmental influences with risk factors for cardiovascular disease. The criterion for lipid metabolism screening at young ages is not widely accepted and possibly follows a universal or directed screening strategy. Additionally, little is known about its long-term effects or possible risk-benefit despite the growing tendency to start pharmacological therapy. Therefore, this study aimed to review the available bibliography on dyslipidemia in pediatric age to present a practical and structured approach to dyslipidemia that focuses on screening, risk stratification for atherosclerotic disease, and therapeutic approach.

## 1. Introduction

The lipoprotein and triglyceride (TG) concentration reflects lipid metabolism that is modulated by genetic and environmental factors [1]. Dyslipidemia can result from an intrinsic, extrinsic, or a combination of genetic predisposition and external factors [1, 2].

Primary dyslipidemias are a heterogeneous group of diseases of genetic, mono, or polygenic etiology, whereas secondary ones result from the association of risk factors with external factors or other pathologies [3].

Dyslipidemias can change the values of total cholesterol (TC), TG, low-density lipoprotein (LDL) cholesterol, or high-density lipoprotein (HDL) cholesterol and appear from childhood to adolescence alone or in association and persist during adult life [4, 5].

Recently, a sustained increase has been determined in the prevalence of dyslipidemias in pediatrics, probably associated with increased overweight prevalence, which promotes other cardiometabolic risk factors, such as high blood pressure (BP), insulin resistance, and endothelial dysfunction [6–9]. All of these factors are involved in atherogenesis that conditions the leading cause of death in adulthood in developed countries [9, 10].

Scientific evidence has supported the relationship between hyperlipidemia at an early age and arterial intima layer changes, which reinforces the hypothesis that atherosclerosis has an early onset, being a chronic and progressive process, both in primary and secondary dyslipidemia [11–16]. Additionally, the presence of atherosclerosis and echographic findings of endothelial dysfunction in children and adolescents with dyslipidemia can predict early CVD [16–18].

Lipids and lipoproteins concentration stabilizes approximately at the age of 2 years and is similar to those observed in young adults [19, 20]. Thus, lipid profile should never be performed before 24 months, even in children with family members with early cardiovascular disease (CVD) [13, 21, 22].

## 2. Lipid Metabolism

Lipoproteins are macromolecular complexes that allow lipid transport in the plasma and are essentially composed of a simple membrane of phospholipids, unesterified cholesterol, and apolipoproteins that surround TG and cholesterol esters. Their different densities and compositions give them different characteristics [21].

In the exogenous pathway, TG, cholesterol, and fatty acids from the diet are absorbed and, together with other fat-soluble compounds, constitute chylomicrons (CMs). They provide TG to tissues (muscle fibers and adipocytes) as an energy source due to lipoprotein lipase (LPL) endothelial action, and the remaining CMs are captured in the liver. A diet rich in polyunsaturated fatty acids *ω*3 and *ω*6 enhances the action of LPL [23, 24].

In the endogenous pathway, tTG and cholesterol esters that are synthesized in the liver are transported again via the bloodstream to the tissues in the form of very-low-density lipoproteins (VLDL), transformed into lower volume and higher density lipoproteins via LPL, and are finally converted to LDL by the hepatic lipase and recaptured via the LDL receptor (LDL-R). The lipoprotein (a) (Lp [a]) is a subtype of LDL with the addition of ApoA, whose elevation is an independent cardiovascular risk factor [25].

Excess cholesterol is then incorporated into HDL and removed from circulation (reverse transport) by esterification by lecithin cholesterol acyltransferase (LCAT), reused at the hepatocytes.

## 3. Pathophysiology

The pathogenesis of atherosclerosis is multifactorial, with genetic and environmental factors plus inflammatory response effects and the interaction of inflammatory markers (C-reactive protein, interleukins, and amyloid A), hemostatic/thrombotic markers (fibrinogen, factors V, VII, and VIII, d-dimers, and tissue plasminogen activator), lipid factors (Lp [a], apolipoproteins A and B, and lipase A2), homocysteine, and angiotensin-converting enzyme [25]. Obesity, high BP, and smoking are some of the leading factors for endothelial dysfunction in atherogenesis [26].

The inflammatory response results from cell activation (T lymphocytes, macrophages, and smooth muscle cells), LDL oxidation, growth factor secretion, procoagulation, and proinflammatory cytokines [27–29]. Subsequently, foam cells are formed (due to oxidized LDL molecules phagocytosis), and the atherosclerotic plaque undergoes fibrosis and eventual calcification [29]. This intra- or extracellular accumulation of lipid scan cause early peripheral neurological or gastrointestinal changes [29].

Values of lipids and lipoproteins in children and adolescents vary according to age and gender. The National Cholesterol Education Program and the Identification and prevention of Dietary and lifestyle-induced health EFfects In Children and infants (IDEFICS) study consider values below the 75^th^ percentile (p75) as acceptable, between the 75^th^ and 95^th^ percentile (p75-p95) as borderline and above the 95^th^ percentile (>p95) as high [12, 30]. The most commonly used reference tables on literature are based on the US-American population; however, tables from European studies were presented. Data were obtained through the IDEFICS study from 2 to 10 years of age (13579 children spread over eight European countries) [12], whereas from HELENA study from 12 to 17 years of age (1076 adolescents from nine European countries) [18]. To note, using these reference values is not yet commonly practiced, nor are the percentiles uniform across studies. However, treatment initiation criteria and the therapeutic goals are defined in absolute values.

The likelihood of subclinical atherosclerosis in adulthood, with practical implications for assessing CVD risk in pediatrics, is commonly predicted with non-HDL cholesterol and the TG/HDL cholesterol ratio [15]. The use of TG/HDL cholesterol ratio as an early marker of atherogenic cardiometabolic risk may institute prompt therapy [16]. Little evidence justified the routine use of non-HDL cholesterol in pediatric age, even though it is an indicator of carotid, middle intima layer thickening in adulthood [14–16].

## 4. Classification

In clinical practice, pathogenesis is differentiated as primary or secondary dyslipidemia. Subdivision can also be according to biochemical changes as isolated increased TC or TG, low isolated levels of HDL cholesterol, and lastly, simultaneously increased TC and TG associated with low levels of HDL cholesterol (mixed or combined) [28, 31].

Dyslipidemia sometimes appears as a synonym for hyperlipidemia; however, hypolipidemias are to be considered, and a TC below p5 may be found in abetalipoproteinemia, familial hypobetalipoproteinemia, or sterol synthesis defects [23, 31].

### 4.1. Primary Dyslipidemia

Primary dyslipidemias (hyperlipidemias) are a heterogeneous group of diseases (Table 1) that is characterized by markedly elevated cholesterol (usually LDL), TG, or rarely, a combination of both [23–25].

#### 4.1.1. Hypertriglyceridemia

Familial hypertriglyceridemia is the result of changes in CM or VLDL usage. LPL comprises 95% of cases, or rarely due to mutations in APOC2, LMF1, GPIHBP1, and APOA5. Familial LPL deficit is characterized by increased TG levels in fasting (>1000 mg/dl), which may cause gastrointestinal symptoms, including severe pancreatitis, without atherosclerosis association or increased cardiovascular risk [32]. Retinal vein injury (retinal lipemia) and milky appearance of the blood plasma, in addition to paresthesia, dyspnea, and confusion, can arise with TG of >2000 mg/dl [33]. Aversion and self-avoidance of foods with a high caloric load can delay clinical manifestations until adulthood [33, 34]. Despite autosomal dominant (AD) transmission, a clear genetic basis is unavailable.

#### 4.1.2. Hypercholesterolemia

Familial hypercholesterolemia is characterized by increased LDL levels due to changes in hepatic LDL-R (familial hypercholesterolemia), changes in the apolipoprotein B100 protein receptor on the LDL particle surfaces (apolipoprotein B-100 deficit), or gain in the proprotein convertase subtilisin/Kexin type 9 function, which is involved in LDL-R degradation [35]. Homozygous forms are rare but present at an early age more severe form (CVD in the first–second decade of life) than heterozygous forms, illustrating a broad phenotypic spectrum [4, 28]. Regardless, the risk of early CVD is significant in both.

Sitosterolemia is caused by excessive plant sterol absorption at the intestinal level, with elevated TC, resulting in xanthomas, premature atherosclerosis, hemolytic anemia, and macrothrombocytopenia [36].

Hepatic lipase hydrolyzes TG and phospholipids in VLDL and intermediate-density lipoprotein remnants prevent their conversion to LDL. Its deficit causes an increased TC, HDL cholesterol, and TG from adolescence [2], associated with increased transaminases, thus high HDL cholesterol levels suggest the diagnosis [37]. Statin treatment in these patients can worsen liver diseases, thereby emphasizing the importance of not indiscriminately initiating treatment [38].

#### 4.1.3. HDL Decrease

Primary HDL metabolism changes are infrequent and include apolipoprotein A-1 deficiency, familial hypoalphalipoproteinemia, Tangier disease, LCAT deficit, and esterified cholesterol transfer protein deficiency [25]. They may cause premature atherosclerosis, neuropathy, nephropathy, and cornea opacity. Low LDL and TG levels can induce symptoms of fat malabsorption, such as steatorrhea, fat-soluble vitamin deficiency, and poor weight progression [39]. Tangier disease has an accumulation of free cholesterol in the reticuloendothelial system that causes orange discoloration of the reticuloendothelial system, which can be easily seen as enlarged and orange tonsils [28].

### 4.2. Combined Dyslipidemia

It is characterized by moderate to severe increased TG levels associated with low HDL levels (with increased non-HDL cholesterol) and is currently the most common form in pediatric age. It results from the epigenetic and environmental influence, that is, the combination of genetic variants and polymorphism effects with external stimuli (diabetes mellitus (DM), obesity, kidney disease, hypothyroidism, high-calorie diet, fats, or alcohol) [3]. It occurs almost exclusively in adolescents with obesity association (30%–60% of young people with obesity) [40, 41].

Genetically complex combined familial hyperlipidemia is characterized by moderately increased LDL and TG levels and reduced HDL levels that are often associated with a family history of early CVD. The definitive diagnosis implies the existence of at least two family members (first degree) with LDL or TG levels of >p90. They may have high levels of ApoB that are associated with metabolic syndrome; however, the presence of xanthomas is not characterized [32].

Familial dysbetalipoproteinemia is related to apolipoprotein-E, a similarly increased TC and TG, with normal HDL. Definitive diagnosis is obtained by lipoprotein electrophoresis (beta band predominance), VLDL: TG ratio of >0.30, or ApoE2 isoform homozygosity. In adulthood, it can present with xanthomas (knees, buttocks, and elbows) and yellow-orange palmar discoloration, but it appears as a nonspecific rash in pediatric age [25, 28, 32].

Polygenic hypercholesterolemia is the most common cause of increased LDL, without a predictable hereditary predisposition but a familial pattern, in which genetic risk factors are reinforced by similar habitual exposure [25, 35].

### 4.3. Secondary Dyslipidemia

**Figure 1:**
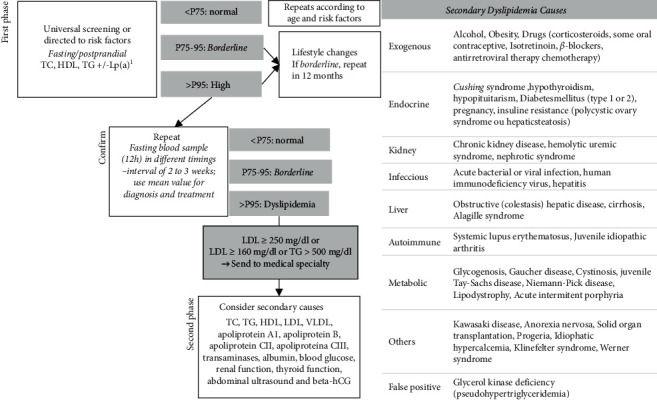
Dyslipidemia screening algorithm and secondary dyslipidemia causes. ^1^Stroke with no identifiable cause, familial hypercholesterolemia, premature cardiovascular disease in 1^st^ or 2^nd^ degree relatives or 1^st^ degree relative with high levels of lipoprotein (a). HDL, High density lipoprotein; LDL, low density lipoprotein; Lp(a), Lipoproteina (a); TC, total cholesterol; TG, triglycerides; VLDL, very low density lipoprotein.

These arise on exogenous factor condition changes in lipid metabolism (see Figure 1) [5, 42], sometimes associated with genetic predisposition (genes with little individual effect). They can be grouped according to the following variation they promote [9–12, 26]:

1. Hypercholesterolemia: hypothyroidism, nephrotic syndrome, cholestasis, anorexia nervosa, and some drugs (progesterone, thiazide diuretics, carbamazepine, and cyclosporine)
2. Hypertriglyceridemia: obesity, type 2 DM, alcohol consumption, kidney failure, sepsis, stress, Cushing's syndrome, pregnancy, hepatitis, human immunodeficiency viral infection, and drugs (protease inhibitors, anabolic corticosteroids, *β*-blockers, estrogen, and thiazide diuretics)
3. Low HDL levels: smoking, physical inactivity, obesity, type 2 DM, malnutrition, steroids, and *β*-blockers

## 5. Diagnostic Approach

### 5.1. Initial Approach

Complete anamnesis includes detailed family history (considered early CVD [angina or acute myocardial infarction, coronary artery bypass graft, angioplasty, stroke, peripheral arterial disease, or sudden cardiac death] if it occurs in females of <65 years or in males of <55 years), pancreatitis history, and secondary dyslipidemia causes [40, 41].

Fat malabsorption signs and symptoms, such as steatorrhea or poor weight progression, should be sought, and anthropometry (body mass index and abdominal perimeter), BP, organomegaly, and presence of xanthomas, xanthelasmas, or corneal arch should be assessed [42, 43].

Opting for a universal screening strategy or one directed to the family or individual risk factors in asymptomatic patients is possible. Selective screening should be performed on children/adolescents over 2 years old with family or individual risk factors (overweight/obesity, hypertension, smoking, sedentary life, or DM) as soon as these factors are identified and regularly while present (with 2 or 3 years interval) [9, 13, 20]. Selective screening criteria will neglect the diagnosis of 30%–60% of children with dyslipidemia; therefore, a universal opportunistic screening strategy should be considered between 9 and 11 years old and after the pubertal stage development (17–21 years old) [5]. According to the NHLBI guidelines, children with ages 12–16 years should not be screened as they can present a falsely low result due to decreased lipid synthesis [34]. Lipid profile must be assessed in all children/adolescents with signs or symptoms of dyslipidemia [1].

**Figure 2:**
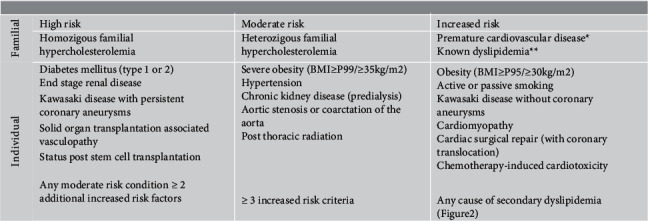
Dyslipidemia familial and individual risk factors. ^*∗*^Familial history (1^st^ and 2^nd^ degrees) of premature cardiovascular disease (male <55 yr; female <65 yr)-angina or acute myocardial infarction, coronary artery bypass graft, angioplasty, stroke, peripheral artery disease, or sudden cardiac death; ^*∗∗*^1^st^ degree relative: TC >240 mg/dl and/or LDL >130 mg/dl and/or TG >170 mg/dl and/or HDL <35 mg/dl.

The diagnostic and therapeutic approach is summarized in Figure 2. Regardless of the screening strategy, the first phase of the dyslipidemia approach is the cardiovascular risk stratification (increased, moderate, or high-risk CVD development in 10 years) (Figure 2) [34]. High-risk conditions are uncommon in childhood, but their identification is crucial [39]. Isolated dyslipidemia (without other risk factors), not per se an absolute criterion for initiating therapy, merely leads one to consider these patients as having a greater CVD risk than the rest of the population [44].

Screening can be at fast or postprandial (higher TG in the latter) but must be confirmed in two fasting samples (12-h minimum fast) if altered, 2–3 weeks apart (see Figure 1). The average between these two values will be used for diagnostic and therapeutic purposes [4]. The postprandial sample implies the determination of non-HDL cholesterol by subtracting HDL from the TC [15]. Inflammation secondary to severe infections can cause significantly increased TG, for which lipid profile screening should not be performed in 3 weeks after infections.

In the first phase, the main fractions (CT, HDL, and TG) should be quantified, and the apolipoprotein B or apolipoprotein A-1 determination is not indicated. LDL is calculated using the Friedewald formula (LDL = TC − HDL − TG/5), except if TG is >400 mg/dl, in which case LDL must be directly measured [4]. People under 20 years old who had a cerebral stroke of unknown etiology, familial hypercholesterolemia, and family history (first or second degree) of premature or familial CVD (first degree) with increased Lp (a) are advised to dose Lp (a) [45]. TC value of ≥250 mg/dl or LDL of ≥160 mg/dl or TG of >500 mg/dl should be referred to a specialist consultation [33, 34].

If dyslipidemia is confirmed in a second fasting sample, a complementary study (second phase) should be conducted to evaluate the remaining fractions: VLDL, apolipoprotein A1, apolipoprotein B, apolipoprotein CII, and apolipoprotein CIII, and possible (co)existence of secondary causes (blood count, blood glucose, HbA1c, creatinine, urea, aspartate [AST], and alanine [ALT] aminotransferases, free thyroxine, and thyroid-stimulating hormone; an upper abdominal ultrasound and, if pertinent, beta-human chorionic gonadotropin) [2, 34].

### 5.2. Differential Diagnosis

The spectrum of presentation may overlap between various entities; however, monogenic primary dyslipidemia should be considered with the following [46, 47].

- Family history of dyslipidemia, tendon xanthomas, or premature CVD
- Family or personal history of recurrent or very early pancreatitis
- Presence of tendon or cutaneous xanthomas
- LDL of >500 mg/dl or TG of >1000 mg/dl (values below are usually polygenic)

Other inherited metabolic diseases lead to the intracellular accumulation of cholesterol without necessarily inducing lipid profile changes:

- Defects in bile acid synthesis (cerebrotendinous xanthomatosis) with increased bile acid metabolism intermediates with cholestanol production
- Lysosomal diseases, such as Niemann-Pick disease type C (lipidosis due to intracellular cholesterol transport defect) or cholesterol ester deposit disease and Wolman's disease (lysosomal acid lipase deficiency)

Other inborn metabolism errors that lead to lipoprotein value changes are suggested by the following [23, 25]:

- Pseudo hypertriglyceridemia (elevated serum glycerol) in glycerol kinase deficiency
- Hypertriglyceridemia (and hyperuricemia) in glycogenosis I
- Combined dyslipidemia (can be severe) in lipodystrophies
- Decreased TC: sterol synthesis defects (e.g., Smith-Lemli-Opitz syndrome) or secondary to congenital protein glycosylation disabilities

## 6. Treatment and Follow-Up

**Figure 3:**
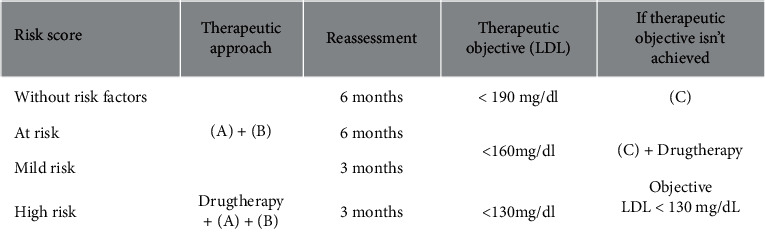
Dyslipidemia therapeutic approach by risk stratification. (A) Lifestyle changes; (B) CHILD-1 Diet; (C) CHILD-2 Diet.

The therapeutic approach and therapeutic goals depend on the risk of developing atherosclerotic CVD, and the criteria are more restricted for those at high risk (Figures 2 and 3) [48, 49]. It aimed to reduce CVD risk in the future; thus, treatment criteria will depend primarily on LDL values.

### 6.1. Dietary Intervention and Lifestyle Changes

The treatment basis is focused on diet and at least 30–60 min of physical activity [1, 48, 49]. Tobacco smoke exposure (passive or active) should be avoided and age-appropriate sleeping habits should be adopted. Additionally, screen time should be limited to <2 h per day since some studies observed that every additional hour was correlated with increased TG and decreased HDL levels [48]. These interventions should be family-centered to optimize their impact and length [16].

Dietary treatment of hypercholesterolemia is not indicated below 2 years of age due to the increased need for dietary fats (rapid growth and development of the nervous system) [42, 48].

The recommended diet is based on increased consumption of fruit, vegetables, and whole grains compared to the percentage of ingested fat (lipids by 25%–30%; carbohydrates by 55%, and proteins by 15%–20% of the total calories) [33].

In an initial phase (CHILD-1 diet), the fat content in the diet must have <10% saturated, ≤1% trans, and <300 mg/day cholesterol [5, 33]. The first prescribed diet is maintained in LDL of <130 mg/dL [5, 33]. Additionally, LDL of ≥130 mg/dL in the 3-month reevaluation will have a more restricted diet (CHILD-2 diet) of lipids by 25% of the total calories with saturated at <7%, trans reduced to a minimum, and cholesterol at <200 mg/day (monitoring by a nutritionist), and pharmacological treatment should be considered (Figure 3).

Initiating supplementation with 1.5–2 g/day of stanols and plant sterols [48, 50], substances structurally similar to cholesterol that inhibit their intestinal absorption [50], naturally present in fruits, vegetables, vegetable oils, nuts, and seeds, is feasible in children older than 6 years [48]. Supplemented commercial products have variable concentrations and can be used as coadjuvants in lowering LDL (∼8% reduced TC) [42, 50].

In hypertriglyceridemia, initial dietary measures include decreased sugar consumption and fish intake (rich in *ω*-3) promotion. Caloric distribution should be total fat of 30%–35% (saturated of <10%, trans of <1%, and cholesterol of <300 mg/day), carbohydrates of 50%–60%, and proteins of 10%–15% of the total calories [5, 33]. Without the expected results, dietary intake should be restricted to a total fat of 25%–30% and total saturated calories of <7% (trans of <1%, corresponding monounsaturated of 10%, and cholesterol of 200 mg/day) [5, 33].

### 6.2. Pharmacological Therapy

Pharmacotherapy should be considered according to CVD risk stratification [16]. The decision to start pharmacological treatment depends on age, severity, and the presence of other individual or familial CVD risk factors (see Figure 2) [16,48]. Lifestyle changes are recommended, with a particular incidence of dietary treatment for 3–6 months, before the pharmacological therapy reassessment and decision-making [16, 42, 48]. Pharmacological treatment may be instituted *ad initium* in high-risk individuals with LDL of ≥130 mg/dl and age of >10 years [36, 48]. In the case of pharmacological therapy indication, the patient should be referred for hospital consultation.

Long-term effectiveness in reducing CVD risk has been proven in familial hypercholesterolemia (homo and heterozygous forms) [36, 39].

The pharmacological options are several and must be selected according to the lipid profile and risk-benefit (Table 2).

Statins inhibit the reductase of 3-hydroxy-3-methylglutaryl-coenzyme-A, an enzyme that limits endogenous cholesterol synthesis with decreased intracellular cholesterol content and increased LDL clearance. They are the first line of treatment (the main objective is LDL of ≤130 mg/dL or at least reduce the baseline value in 50%), are recommended from 8 years of age, and are contraindicated in pregnancy (teratogenic risk); thus, the use in adolescents/women of childbearing age must be associated with contraception [42, 48, 51]. The most commonly used are rosuvastatin or pravastatin (over 8 years old), and other statins (atorvastatin, simvastatin, or lovastatin) are recommended above 10 years old [48]. Studies advise taking it at bedtime with mandatory clinical and analytical monitoring at 4, 8, and, if necessary, 12 weeks of treatment (lipid profile, AST, ALT, HbA1c, and creatinine kinase) [42, 48].

Bile acid scavengers (e.g., cholestyramine) bind to bile acids, reducing their absorption, and increasing their hepatic synthesis, thus decreasing the cholesterol content of the hepatocytes. They can be used in over 6-year-olds, in monotherapy, or with statins but are not recommended with TG of >500 mg/dL, whereas carefully prescribed in >250 mg/dL [48]. They are rarely used because they limit the absorption of fat-soluble vitamins and some drugs because of their side effects (abdominal pain and diarrhea) and are less effective than statins [42, 48].

Cholesterol absorption inhibitors (e.g., ezetimibe) inhibit intestinal cholesterol absorption from plant sterols. They can be used from 10 years of age as monotherapy or in association with statins, useful in children/adolescents with familial hypercholesterolemia or high-risk factors for premature CVD, who do not reach therapeutic goals with the optimized statin dose. They do not alter TG, vitamin A, and D, fat, or bile acids absorption [42, 48].

Fibrates are agonists of nuclear PPAR-*α* receptors and favor TG and VLDL degradation [42]. They are preferentially used in hypertriglyceridemia, but their use in under 18 years old is not yet approved, thus only indicated in children with hypertriglyceridemia of >500 mg/dl or at risk of pancreatitis, who are unresponsive to dietary measures [5, 34, 48]. The simultaneous use of statins enhanced adverse muscle effects [34, 42, 48].

### 6.3. Treatment Specificities

Drugs for hypertriglyceridemia are ineffective, with a low-fat diet as the only effective therapy in familial LPL deficit. The *ω*-3 fatty acids (decrease hepatic fatty acid content, TG synthesis, and VLDL release) may play a role in hypertriglyceridemia treatment [42]. Homozygous forms of hypercholesterolemia usually poorly respond to standard treatment options, even with the maximum optimized doses. Therefore, lipoprotein apheresis is an additional option for these patients; however, its effect on LDL concentrations is temporary and should be performed ideally every 1–2 weeks. This treatment should be started as soon as possible, and it is recommended as an option from age of 5 years and initiated before the age of 8 years [48].

The main point of the therapeutic approach in combined family hyperlipidemia involves greater fruit and vegetable consumption and moderate physical activity for at least 1 hour per day, 5 days a week, coupled with reduced consumption of sugary foods and drinks [34, 41].

### 6.4. Novel Treatment Options

Recently, novel lipid-lowering drugs (evolocumab, alirocumab, evinacumab, and mipomersen) were described in several studies. Currently, they are approved for patients with severe familial hypercholesterolemia above the age of 12 years and they are trial in children to extend their recommendation to other ages and entities. Drugs, such as Lomitapide, Bempedoic acid, or Inclisiram, are still under evaluation and do not have robust evidence that supports their use in pediatric patients [48].

## 7. Conclusions

Atherosclerosis is a dynamic and inflammatory process; however, it can be modified, which highlights the importance of early intervention. Dyslipidemia screening at the pediatric age assumes that early identification and treatment will reduce cardiovascular risk in adulthood. Treatment effectiveness in the pediatric age was derived from studies on children with familial hypercholesterolemia, in which the atherosclerotic process control has been demonstrated [48]. However, high cholesterol levels have a multifactorial origin in the majority of cases, and the risk increase for CVD remains unknown. Therefore, the diagnostic and therapeutic approach to dyslipidemia in the pediatric age is a significant challenge. Therapeutic cut-offs and goals are based on CVD risk assessment in adulthood, and studies in children and adults reveal a safe profile, although the long-term effects of statin therapy are uncertain.

## Figures and Tables

**Table 1:** Primary dyslipidemias and their main characteristics (adapted from [25, 28, 35, 47]).

Disease	Gene, heredity prevalence	Phenotype	Lipid profile			
			TG	TC	HDL	Others
Exogenous pathway (↑↑↑ TG)						
Familial lipoprotein lipase deficiency	LPL	Abdominal pain, pancreatitis, xanthomas, *lipemia retinalis*	↑↑↑ >1000 mg/dl	↑	↓	↑↑↑ VLDL
	Homozygous: 1/10^6^					
	Heterozygous: 1/500					
Apolipoprotein C-II deficiency	APOC2 20 families	Abdominal pain, pancreatitis, xanthomas, *lipemia retinalis*	↑↑↑	↑	↓	
Endogenous pathway (↑↑ TG)						
Familial hypertriglyceridemia	LDL receptor, Apo B100, PCSK9	Abdominal pain, pancreatitis, xanthomas	↑↑ 250–1000 mg/dl		N	Normal Apo-B
	ADI					
	1/500					
Familial combined hyperlipidemia (FCH)	ADI	Hypertension, obesity, insulin resistance, vascular stenosis, cardiovascular disease <50-years	N/↑	↑	↓	↑VLDL
	Homozygous: 3–5/1000					
	Heterozygous: 1/1000					
Familial hypercholesterolemia	LDLR (ADI)	Xanthomas, vascular stenosis, cardiovascular disease <50-years; in homozygous, premature myocardial infarction, corneal arcus	↑↑ ≥1000 mg/dl		↑↑ ≥600 mg/dl	↑↑ ApoB Heterozygous: ↑ 270–550 mg/dl), LDL (≥160 mg/dl)
	Homozygous: 1/1.6-3x10^5^					
	Heterozygous 1/270					
Exogenous and endogenous pathways (↓LDL)						
Abetalipoproteinemia	MTTP (ARI) <1/10^6^	Malabsorption, ataxia, pigmentary retinopathy	↓↓	↓	↓	↓↓LDL, ↓↓Apo B (↑prothrombin time)
Hypobetalipoproteinemia	ADI	Malabsorption, ataxia, pigmentary retinopathy	↓↓	↓↓		↓↓ LDL
Familial dysbetalipoproteinemia (ApoE)	Apo-E (ARI) 0.5/1000	Xanthomas, vascular stenosis, cardiovascular disease <50-years	↑250–600 mg/dl	↑250–500 mg/dl	N	VLDL/TG >0.3 and cholesterol ratio TC/TG >0.42
Exogenous and endogenous pathways (↑ TG and ↑ TC)						
Lipase deficiency	LIPC (ARI) 7 families	Hepatomegaly, cardiovascular disease <50-years	N/↑	N/↑	N/↑	Normal TC, HDL and TG in pediatric age but ↑ in adulthood
Apolipoprotein B-100 deficiency	Apo-B100 (ADI)	Xanthomas, vascular stenosis, cardiovascular disease <50-years	N	↑	↓/N	↑ LDL and Apo B
	Homozygous: 1/4 × 10^6^					
	Heterozygous: 1/1000					
	PSCK9 (ADI)	Xanthomas, coronary disease, premature myocardial infarction	N	↑↑	↓	
Reverse cholesterol transport pathway changes (↓HDL)						
Familial hypoalphalipoproteinemia	Apo-AI (ADI/ARI)	Increased risk of cardiovascular diseases	N	N	↓↓	
	Unknown					
Apolipoprotein A-I mutations	APOA1 (ADI)	Vascular stenosis, cardiovascular disease <50-years	N	↓↓	↓↓	↓↓ ApoA1
	<1/10^6^					
Tangier disease	ABCA1 (ARI)	Orange tonsils, splenomegaly, premature atherosclerosis	N/↑	↓↓	↓↓ <5 mg/dl	↓↓ Apo A1 (<30 mg/dl)
	100 reported cases					
Lethicin-cholesterol acyltransferase deficiency (LCAT)	LCAT (ARI)	Corneal arcus, corneal deposits <20-year-old; if total deficiency it can leads to renal failure	↑	N	↓↓	↓↓ ApoA1
	125 reported cases					
Exogenous factors						
Polygenic hypercholesterolemia	? (PG) 2–5/1000	Increased risk of cardiovascular diseases xanthelasma (not xanthomas) and corneal arcus may be present	↑	↑/↑↑	↓/↓↓	↑/↑↑ LDL

ADI, autosomal dominant inheritance; APO, apolipoprotein; ARI, autosomal recessive inheritance; HDL, high density lipoprotein; LDL, low density lipoprotein; LDLR, low density lipoprotein receptor; LPL, lipase lipoprotein; PG, polygenic; TC, total cholesterol; TG, triglycerides; VLDL, very low density lipoprotein.

**Table 2:** Drug dosage for pediatric dyslipidemia treatment (adapted from [48]).

Class	Cholesterol reduction (%)	Initial dosage (mg/day)	Maximum dose (mg/day) >10 years (maximum in adulthood)	Side effects
Statins				
Atorvastatin	40–45	5–10	20 (80)	Myopathy elevation of liver enzymes
Lovastatin	21–36	10	40 (80)	
Pravastatin	23–33	5	20 (8–14 yr); 40 (>14 yr) (80)	
Rosuvastatin	28–50	5	20	
Simvastatin	17–41	5 (10 yr) 10 (>10 yr)	40 (40) 10 (10)	
Cholesterol absorption inhibitors				
Ezetimibe	18	10		Abdominal pain, diarrhea, flatulence, the elevation of liver enzymes and creatine kinase
Bile acid sequestrants				
Cholestyramine	12	2000–4000	8000 (16 g)	Meteorism and constipation; inhibition of fat-soluble vitamins absorption
Fibric acid derivates (fibrates)				
Gemfibrozil	18	600–1200	No data available (1200)	Muscle pain/weakness, with liver enzymes and creatine kinase elevation
Fenofibrate	22	40	No data available (130–200)	
Omega-3 fatty acids				
Omega-3 ethyl esters		1000	No data available (4 g)	

## Data Availability

No data were used.
